# Quantitative PCR assay for the detection of *Aedes vigilax* in mosquito trap collections containing large numbers of morphologically similar species and phylogenetic analysis of specimens collected in Victoria, Australia

**DOI:** 10.1186/s13071-021-04923-y

**Published:** 2021-08-28

**Authors:** Peter T. Mee, Shani Wong, Karen Brown, Stacey E. Lynch

**Affiliations:** Agriculture Victoria Research, AgriBio Centre for AgriBioscience, Bundoora, Victoria Australia

**Keywords:** *Aedes vigilax*, qPCR, Whole trap processing, Phylogenetics

## Abstract

**Background:**

*Aedes vigilax* is one of the most significant arbovirus vector and pest species in Australia’s coastal regions. Occurring in multiple countries, this mosquito species occurs as a species complex which has been separated into three clades with two detected in Australia. Until recently, *Ae. vigilax* has largely been absent from Victoria, only occasionally caught over the years, with no reported detections from 2010 to 2016. Complicating the detection of *Ae. vigilax* is the shared sympatric distribution to the morphologically similar *Ae. camptorhynchus*, which can exceed 10,000 mosquitoes in a single trap night in Victoria. Currently, there are no molecular assays available for the detection of *Ae. vigilax*. We aim to develop a quantitative PCR (qPCR) for the detection of *Ae. vigilax*, with the specificity and sensitivity of this assay assessed as well as a method to process whole mosquito traps.

**Methods:**

Trapping was performed during the 2017–2020 mosquito season in Victoria in two coastal areas across these 3 consecutive years. A qPCR assay was designed to allow rapid identification of *Ae. vigilax* as well as a whole mosquito trap homogenizing and processing methodology. Phylogenetic analysis was performed to determine which clade *Ae. vigilax* from Victoria was closest to.

**Results:**

*Aedes vigilax* was successfully detected each year across two coastal areas of Victoria, confirming the presence of this species. The qPCR assay was proven to be sensitive and specific to *Ae. vigilax*, with trap sizes up to 1000 mosquitoes showing no inhibition in detection sensitivity. Phylogenetic analysis revealed that *Ae. vigilax* from Victoria is associated with clade III, showing high sequence similarity to those previously collected in New South Wales, Queensland and Western Australia.

**Conclusions:**

*Aedes vigilax* is a significant vector species that shares an overlapping distribution to the morphologically similar *Ae. camptorhynchus*, making detection difficult. Here, we have outlined the implementation of a specific and sensitive molecular screening assay coupled with a method to process samples for detection of *Ae. vigilax* in collections with large numbers of non-target species.

**Graphical abstract:**

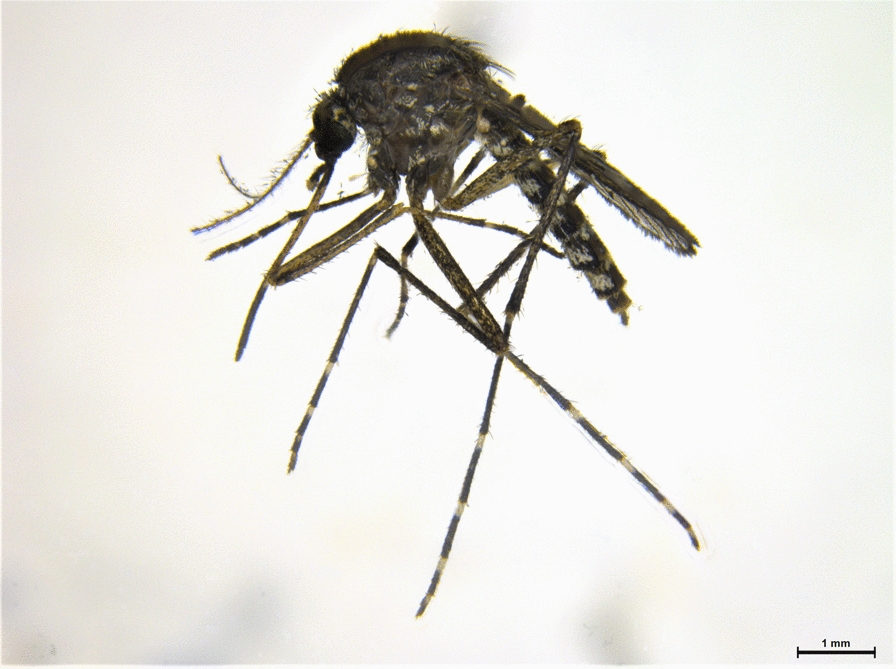

**Supplementary Information:**

The online version contains supplementary material available at 10.1186/s13071-021-04923-y.

## Background

*Aedes vigilax* is regarded as an aggressive mosquito species, with a host preference for humans, other mammals and occasionally avian species [1, 2]. *Aedes vigilax* is regarded as the principal vector of Ross River virus (RRV) in coastal and sub-coastal areas of Australia [3, 4] as well as vectoring a range of other arboviruses such as Barmah Forest virus (BFV) and has experimentally been proven competent to exotic arboviruses such as chikungunya virus (CHIKV), West Nile virus and Japanese encephalitis virus [3–5]. Active during crepuscular periods, *Ae. vigilax* can bite during the day, particularly around larval habitats where large populations can occur. This mosquito species has been classified as a diverse generalist feeder increasing this mosquitoe species’ ability to potentially act as a bridge vector of a range of pathogens [2].

*Aedes vigilax* (known as the northern saltmarsh mosquito) breeds episodically in coastal regions of Australia, with the female depositing her eggs in damp soil associated with floodplains, mudflats and brackish to hypersaline pools, with a high density of eggs occurring at vegetated sites amongst mangroves and artificial drainage areas [6, 7]. These coastal sites get flooded by above average or “king” tides causing large hatching events of this species [8]. *Aedes vigilax* populations can sporadically explode (with overnight collections in the 1000s) with the coincidence of high tides, warmer air temperatures and day length [6]. Along Australia’s coastline, *Ae. vigilax* occurs in New South Wales, Queensland, Northern Territory, Western Australia and South Australia and has generally been thought to be absent or not well established in Victoria and Tasmania [9, 10]. Although first reported in the northwest of Victoria in the original surveys by Lee et al. in 1984, there have only been sporadic detections of this species in the state-wide mosquito and arbovirus surveillance program (the Victorian Arbovirus Disease Control Program). More recently, two individuals were detected in 2005 in the Bass coast, a single individual in 2008 in Attwood, 17 in Wellington in 2009 and 8 individuals in Moira 2010 [11, 12], with this representing the last detection of this species until the recent 2017 trapping reported here.

*Aedes camptorhynchus* (known as the southern saltmarsh mosquito) has a similar habitat to *Ae. vigilax* and a sympatric distribution but prefers lower mean temperatures and is common in the coastal areas of Victoria and other parts of southern Australia [6]. Outbreaks of Ross River virus (RRV) [13–15] and Barmah Forest virus (BFV) [16] have been associated with a high abundance of this mosquito species. Previous records have indicated a potentially more extensive geographic distribution of *Ae. camptorhynchus*, with reported detections in non-coastal sites such as inland regions with high salinity, including Mildura in Northern Victoria and the Wheatbelt Valleys in Western Australia [17–20]. Morphologically, these two species are similar, apart from a few distinguishing characteristics such as pale scales on the wings and hook-shaped scales on the *Ae. vigilax* tergites [21], which can be easily missed if the specimens are damaged or in large collections. In addition to the morphological similarities to other species, *Ae. vigilax* occurs as a complex of three clades with morphological and molecular variations, with two of these occurring in Australia [22–24]. Variation in vector competence has been detected between *Ae. vigilax* populations' ability to transmit viruses and filarial parasites, with these variations possibly reflecting differences between clades [23, 25].

Molecular assays, such as conventional PCR [26] or PCR-restriction fragment length polymorphism (RFLP) [27], have widely been used to identify significant mosquito species. However, these techniques require a time-consuming visualization process after amplification and are not suited for rapid processing of traps containing large numbers of mosquitoes. The use of species-specific qPCR assays have been implemented to rapidly and effectively identify exotic species [28, 29] and inform public health risk assessments for arboviral diseases [30]. Sequencing-based detection of mosquitoes and the viruses they transmit in recent years has increased in popularity [31, 32]. However, in many cases, this can result in reduced sensitivity compared to qPCR due to the non-specific nature of these techniques and the need for higher quality DNA due to the larger fragments being targeted [29, 33, 34]. Methods that are based on the processing of whole mosquito traps can also have compromised detection sensitivity as a result of the presences of PCR inhibitors; however, rigorous method development for whole mosquito trap processing can overcome these inhibitors, as has been documented.

During the 2017/2018 mosquito season, *Ae. vigilax* was first detected in the saltmarsh areas of the Gippsland Lakes in Victoria, Australia, a region historically dominated by *Ae. camptorhynchus*. These detections led to expanded surveillance to understand the distribution of *Ae. vigilax* and determine whether this species had established in the area. Here, we present the development of a specific qPCR assay and a whole trap processing method that can be used to efficiently detect *Ae. vigilax* in whole trap collections. The phylogenetic relationship of the newly detected *Ae. vigilax,* with established *Ae. vigilax* clades was also investigated by sequencing three loci, one mitochondrial cytochrome c oxidase subunit 1 (*COI*) [35] and two nuclear genes, alpha amylase and the zinc finger gene [23]. This investigation provides further understanding of the occurrence of *Ae. vigilax* in Victoria as well as the development of a new molecular method for the detection of individual mosquitoes in whole traps.

## Methods

### Mosquito trapping

**Fig. 1:**
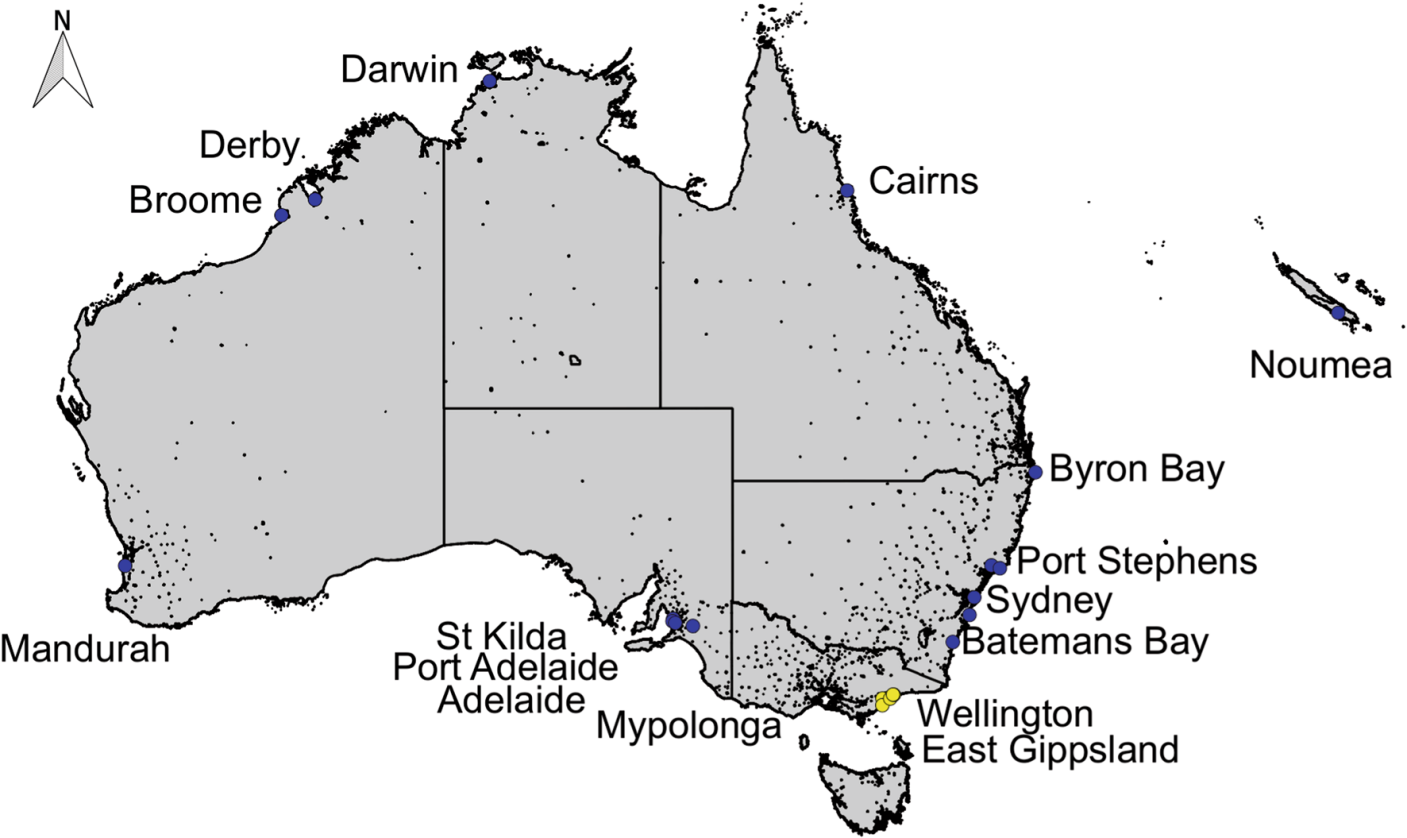
Map of Australia and New Caledonia with dots representing *Ae. vigilax* collection sites, with sequences of individuals for each location included in the *Ae. vigilax* phylogenetic analysis. Yellow dots indicating *Ae. vigilax* collection sites for this study. Blue dots indicating sites from previous studies [23]. Trap locations include Victoria, East Gippsland (EAS) and Wellington (WEL); South Australia, St Kilda (SK), Port Adelaide (PA), Adelaide (AD) and Mypolonga (MY); New South Wales, Byron Bay (BY), Port Stephens (PS), Sydney (SY), Shellharbour (SH) and Batemans Bay (BA); Queensland, Cairns (CA); Northern Territory, Darwin (DA); Western Australia, Derby (DE), Broome (BR), Goegrup Lake (GL) and Mandurah (MA); New Caledonia, Noumea (NO). The map was created in QGIS v2.18.20, using ESRI Shapefiles for Australia [70] and New Caledonia [71]

Mosquitoes were trapped using encephalitis virus surveillance (EVS) traps baited with dry ice pellets as a source of carbon dioxide [36]. The traps were set as part of the Victoria Arbovirus Disease Control Program, a Department of Health and Human Services funded program that supports local government to carry out larval and adult mosquito surveillance and vector control [37]. Traps were set once a week before dusk and collected after dawn, between September to April each year from 2017 to 2020 at a series of sites across Wellington Shire Council and East Gippsland Shire Council, Victoria, Australia (Fig. 1, Additional file 1: Table S1). After collection, mosquitoes were anesthetized by placing the catch bag into an esky with dry-ice for 30 min before transferring to a Petri dish. Petri dishes were sent to the laboratory by express post and on wet ice. Once received at the laboratory, samples were maintained at – 20 °C before processing. All mosquito samples tested in this study were field collected and stored under the same condition as are standard surveillance samples and hence are representative of the condition and quality of these samples. Insects were morphologically identified on a pre-chilled cold bench to preserve the quality of the specimens using a stereo-dissecting microscope and taxonomic keys [21, 38, 39].

### Primers and probe design

*Aedes vigilax* primer and probes were designed using published sequences available in GenBank (JN228453-506, GQ143720-32 and MG242526), which included *Ae. vigilax* from each of the three clades and sequences generated in this study (GenBank: MW542561-71) from individuals collected in Victoria. Sequences were aligned using Mega v7.0.26 before trimming to a conserved region and exporting the alignment for primer selection [40]. PrimerHunter v1.0.2 was used to design the forward Vig_F_S1_9–5′–TTATCCCCTTTGTCATCTG–3′ and reverse Vig_R_S1_23–5′–AAGTAATTCCAGCAGATCGT–3′ primers targeting the *COI* with default parameters except for an optimal amplicon size of < 150 bp [41]. The probe was designed manually and in association with the PrimerHunter software, with a five prime FAM dye and a BHQ1 quencher, 5′- FAM-CATGCAGGAGCTTCAGTAG-BHQ1- 3′ [41]. The final amplicon size procedure by the assay was 149 bp. The designed primers and probe ability to detect all three clades of *Ae. vigilax* from multiple regions were also assessed by performing an *in silico* analysis. Primers and probe were aligned to the sequences outlined above in Mega v7.0.26 and analyzed for their ability to detect all three *Ae. vigilax* clades; in total, 5 sequences from clade I, 16 from clade II and 45 from clade III were compared.

### *Aedes vigilax* qPCR assay parameters

qPCR primers and probe were optimized for concentration using *Ae. vigilax* extracted DNA over a ten-fold dilution series, with an optimal concentration of 400 nM for both primers (Sigma-Aldrich) and probe (Macrogen) determined. *Aedes vigilax* qPCR reactions were performed using TaqMan^TM^ Fast Universal PCR Master Mix (2X), no AmpErase^TM^ UNG (Applied Biosystems), with 10 µl of TaqMan 2X Universal PCR Master Mix, 1 µl of the primer-probe mix and 2.5 µl of DNA, made up to a total reaction volume of 20 µl with nuclease-free water. Inhibition in the qPCR reaction was assessed by adding 2 µl of VetMax Xeno Internal Positive Control DNA (Applied Biosystems) to each reaction. Detection of the Xeno positive control was performed as per the manufacturer’s instruction with the VetMax Xeno Internal Positive Control-VIC Assay (Applied Biosystems). No temple control was added to every qPCR run to assess for contamination and cross primer-dimer formation. The *Ae. vigilax* qPCR was performed on a QuantStudio 5 Real-Time PCR System (Applied Biosystems), with cycling conditions as follows: an initial denaturation at 95 °C for 20 s and 45 cycles at 95 °C for 10 s and 60 °C for 30 s. All qPCR reactions were performed in triplicate. Positives were classified as reactions that produced a cycle quantification (Cq) value < 38, equivocal results were classified between 38 and 40, and negative results of Cq 40 or greater. All data were analyzed using the QuantStudio^TM^ Design and Analysis Software v1.4.3 with the Delta Rn threshold set at 0.05.

### Mosquito pool preparation and processing

The mosquito pools used to assess the sensitivity of the *Ae. vigilax* assay were sourced from Wood Pile, Wellington, and consisted of *Ae. camptorhynchus* (98%) and *Anopheles annulipes* (*s.l.*) (2%). Mosquito pools were prepared by determining the weight of 50 mosquitoes, counted individually and then extrapolating the weight to obtain the required mosquito pools sizes of 199, 399, 599, 799 and 999. Pools were homogenized in 50 ml conical tubes containing a single 9.5-mm stainless steel grinding bead with 2 ml of MEM medium added (8% FBS, 0.1% amphotericin, 1% antibiotics [penicillin and streptomycin], 10% L-glutamine and 1% HEPES) per 100 mosquitoes. Homogenization was performed using a 2010 Geno/Grinder (Thomas Scientific) automated tissue homogenizer at two cycles of 1000 strokes/min for 1.5 min, with the samples kept on ice between cycles. An 80 µl aliquot was taken from each pool and tested in triplicate with the *Ae. vigilax* qPCR to confirm the absence of this species. Pools were subsequently spiked with a single *Ae. vigilax* and homogenized again as outlined above. A single *Ae. vigilax* in a volume of 20 ml of media was included as a positive control, representing the amount of media added to the maximum pool size (1000 mosquitoes) to assess the diluting factor of the media and any inhibitors present in the mosquito pools. Homogenized samples were clarified using a double centrifuge method: first, at 2000 g for 15 min, with the supernatant transferred into a clean tube, and then centrifuged for another 2000 g for 5 min.

### DNA extraction

Eighty microliters of supernatant was extracted using a DNeasy Blood and Tissue kit (Qiagen) following the insect protocol. Homogenates were spiked with 2 µl of VetMax Xeno Internal Positive Control DNA (Applied Biosystems) to assess inhibition. Homogenates were incubated for 1 h with 180 µl of buffer ATL and 20 µl of Proteinase K at 56 °C before completing the extraction protocol as per the manufacturer’s instructions.

Individual *Ae. vigilax* for the phylogenetic analysis, qPCR efficiency and the 20 mosquito species used in the *Ae. vigilax* qPCR specificity study was extracted using the ISOLATE II Genomic DNA kit (Bioline). Individuals were incubated in 180 µl of Lysis Buffer GL and 25 µl of Proteinase K at 56 °C for 3 h before removing the individual and completing extraction protocol as per the manufacturer’s instructions, with the exception of the elution performed using 40 µl of preheated (70 °C) elution buffer G.

### *Aedes vigilax* qPCR analytical specificity and analytical sensitivity

To assess the analytical specificity of the *Ae. vigilax* qPCR assay, a selection of the 20 mosquito species frequently detected in the region covering five genera were tested (*Ae. alternans*, *Ae. bancroftianus*, *Ae. camptorhynchus*, *Ae. clelandi*, *Ae. flavifrons*, *Ae. imperfectus* Dobrotworsky, *Ae. notoscriptus*, *Ae. rubrithorax*, *Ae. sagax*, *Ae. theobaldi*, *Ae. vittiger*, *Anopheles annulipes*, *Coquillettidia linealis*, *Cx. annulirostris*, *Cx. australicus* Dobrotworsky & Drummond, *Cx. cylindricus*, *Cx. globocoxitus* Dobrotworsky, *Cx. molestus*, *Cx. quinquefasciatus* Say and *Tripteroides atripes*. Analytical sensitivity of the *Ae. vigilax* assay was assessed by screening mosquito pools with increasing numbers of other mosquitoes (200, 400, 600, 800, 1000) which had a single *Ae. vigilax* added to them. Each mosquito pool was homogenized as outlined above, with three subsamples removed, extracted and tested with the qPCR in triplicate.

The efficiency of the qPCR assay was assessed by generating a standard curve with six ten-fold serial dilutions of an extracted *Ae. vigilax* individual. The *Ae. vigilax* individual was extracted as outlined above, with the DNA concentration determined by testing on a dsDNA HS Assay Kit on a Qubit^TM^ 2.0 (Invitrogen) fluorometer. A starting DNA concentration of 2.08 ng/µl was determined, with ten-fold serial dilutions performed in EB buffer (Qiagen), with the dynamic range of 2.08E^-1^ ng/µl to 2.08E^-6^ ng/µl being tested; 2.5 µl of each dilution was tested four times using the reaction setup outlined above, with a linear regression fit to the replicates. Data were analyzed in RStudio v4.0.2 [42] using the ggplot2 v3.21 and ggmisc [43] packages. The qPCR assay efficiency was derived from the slope of the standard curve, using the following equation E = − 1 + 10^(-1/slope)^.

### Phylogenetic analysis and haplotype networks

Phylogenetic analysis of *Ae. vigilax* was performed by amplifying the mitochondrial cytochrome c oxidase subunit 1 [35] and the two nuclear genes alpha amylase [23] and zinc finger [23]. Amplification was performed using MyTaq HS DNA Polymerase (Bioline), with 5 µl of 5 × MyTaq Reaction Buffer, 1 µl of the respective forward and reverse primer at 10 µM each, 0.5 µl of MyTaq HS DNA Polymerase and 5 µl of DNA with the reactions made up to 25 µl with nuclease-free water. Cycling conditions were as follows: 95 °C for 1 min, 40 cycles at 95 °C for 15 s, annealing at 49 °C, 56 °C or 56 °C for *COI*, alpha amylase or zinc finger, respectively, and extension at 72 °C for 10 s before a final extension at 72 °C for 2 min. PCR products were purified using the ISOLATE II PCR and Gel Kit (Bioline) before capillary sequencing using both the forward and reverse PCR primers for each gene.

Alignment of *Ae. vigilax* sequences from this and other studies [23] was performed using ClustalW in Mega v7.0.26 and trimmed to a consensus region of 591, 828 and 786 bp for *COI*, alpha amylase and zinc finger gene, respectively. Consensus regions for each gene were analyzed using jModelTest2 v2.1.10, topology taking the best of nearest neighbor interchange, subtree pruning and regrafting [44]. Akaike information criterion was used to select the most appropriate substitution model. Maximum-likelihood trees were constructed in PhyML v3.3.2 with 1000 bootstrap replicates; the proportion of gamma distribution and invariable sites were both estimated [45]. The general time-reversible (GTR) model was selected for all trees. Phylogenetic relationships of *Ae. vigilax* were further investigated through the construction of haplotype networks. Statistical parsimony networks were constructed in PopArt using the 591-bp region of *COI* with 95% connection limits in TCS 1.21 [46]. DnasSP v5 [47] was used to investigate the number of polymorphic sites, haplotype and nucleotide diversity. DnasSP was used to test for population expansion using the neutrality tests Tajima’s *D* [48] and Fu’s *F*s [49]. Sequence data accession number can be found in Additional file 2: Table S2 and Additional file 3: Table S3.

## Results

### Repeat detection of *Ae. vigilax* between 2017 and 2020 season

**Fig. 2:**
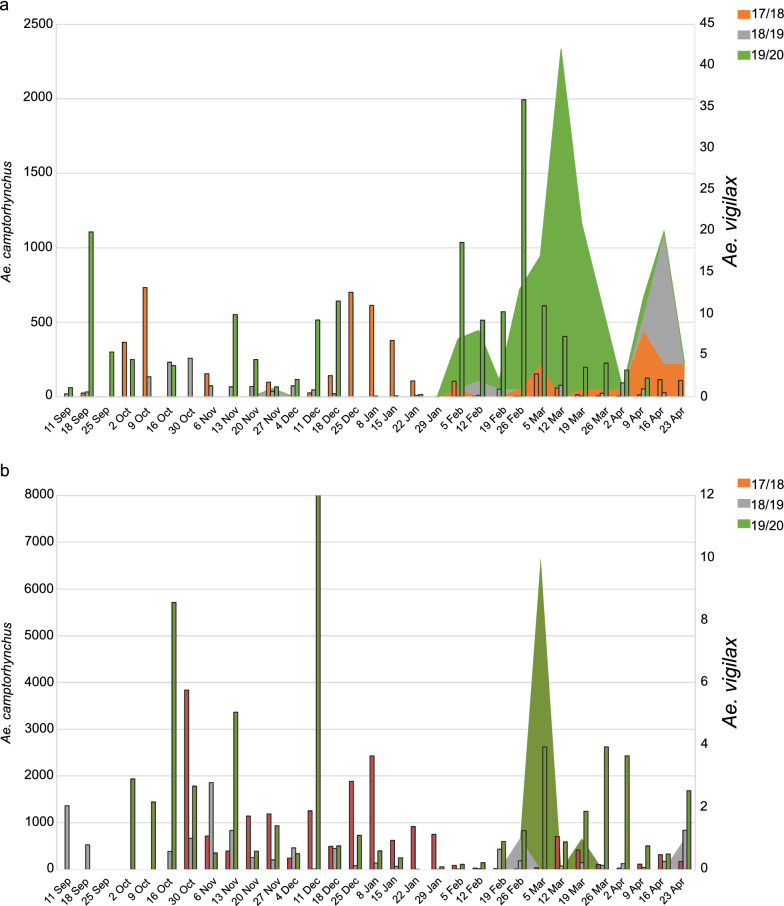
The occurrence of *Ae. vigilax* (stacked area chart) and *Ae. camptorhynchus* (bar chart) at one trapping site in East Gippsland (**A**) shire and one trapping site in Wellington (**B**) shire. Mosquito abundance for *Ae. camptorhynchus* is shown on the left axis and *Ae. vigilax* on the right axis. Trapping events consist of a single trap night with mosquitoes morphologically identified. Mosquito seasons are colored for 2017/2018 (orange), 2018/2019 (gray) and 2019/2020 (green)

Mosquito trapping over 2017 and 2020 identified the presence of *Ae. vigilax* in the East Gippsland and Wellington Shire councils (Fig. 1). The rediscovery of *Ae. vigilax* in these councils in recent years indicates the possible establishment of this mosquito species. Trapping records from 2017 to 2020 showed the maintained presences of this species in both regions (Fig. 2). Peak numbers of *Ae. vigilax* collected during a single catch night was 42 individuals from East Gippsland during the 2019–2020 season, with the largest catch night occurring around March of each trapping season. *Aedes camptorhynchus* numbers were higher than those of *Ae. vigilax* during the annual trapping period with peak numbers reaching 8452 in a single night with a mean trap collection of 204 (range: 0–1993) and 836 (range: 0–8452) individuals from East Gippsland and Wellington, respectively, collected (Fig. 2).

### Development and analytical specificity of the *Ae. vigilax* assay

**Fig. 3:**
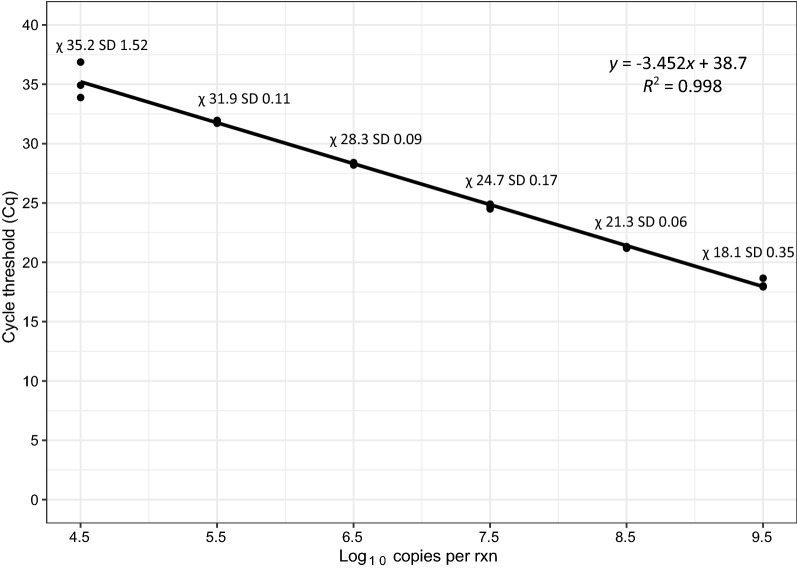
*Aedes vigilax* qPCR efficiency based on four replicates over six ten-fold dilutions. The R^2^ of the regression line is noted on the graph. Mean (χ) and standard deviation (SD) of the four replicates are displayed on the graph

Primers and probes were successfully designed and optimized to detect the presences of *Ae. vigilax*. The qPCR amplification efficiency of the *Ae. vigilax* assay was assessed and determined to have a good level of efficiency at 94.9% over six ten-fold dilution series, with the mean and standard deviation of each dilution displayed (Fig. 3). The assay was successful at detecting the sixth ten-fold dilution with a mean Cq value of 35.2 (log_10_ = 4.5 copies/reaction(rxn)); however, with a seventh dilution (log_10_ = 3.5 copies/rxn) (data not shown), not all replicates were detected indicating the limit of detection for the assay. The analytical specificity of the assay indicated that it is specific to *Ae. vigilax* with no cross-reaction detected between the 20 species tested covering five genera of mosquitoes. No amplification was detected in the no template controls. As no specimens from clade I or II were tested in this study, an *in silico* sequence analysis was performed with the designed primers and probe. Primers and probes were aligned to sequences covering individuals in all three clades and from multiple different regions. A maximum of two nucleotide mismatches were observed across the primers/probe regions when comparing individual sequences across the three clades and never more than one mismatch in the probe sequence.

### Analytical sensitivity and assessment of the whole trap extraction technique using a Xeno internal control

The whole trap mosquito processing method and detection of *Ae. vigilax* were optimized and assessed for inhibition based on Xeno internal control spikes (Table 1). The homogenization of mosquitoes in 2 ml of media per 100 mosquitoes was determined to be optimal and resulted in no significant (ANOVA: *F*(_5, 48_) = 1.41, *P* = 0.23) detectable inhibition with increasing pool size (Table 1). The qPCR was shown to be effective in the detection of a single *Ae. vigilax* in the extrapolated mosquito pool sizes of 200, 400, 600, 800 and 1000 (Table 1). Three aliquots of each pool were processed and screened; each aliquot repeated in triplicates for qPCR testing with an average Cq value of 31.6 ± SD 2 observed across the five pool sizes. There was no significant difference in the Cq values of *Ae. vigilax* in the 200, 400 or 800 pool size (ANOVA: *F*_(2, 24)_ = 0.37, *P* = 0.69) (Table 1). However, pool sizes of 600 and 1000 were three Cq values higher than the smaller pool sizes.

**Table. 1:** qPCR detection of *Ae. vigilax* in mosquito pools

		Mosquito pool size																	
		1			200			400			600			800			1000		
*Ae. vigilax*	Log_10_ copies/rxn	6.695	6.800	6.479	6.174	5.517	6.150	6.092	5.493	5.918	4.864	5.160	4.873	6.008	5.985	5.959	4.745	4.899	5.070
	Mean Cq	27.64	27.28	28.39	29.44	31.71	29.52	29.72	31.79	30.32	33.96	32.94	33.93	30.01	30.09	30.18	34.37	33.84	33.25
	SD of Cq	0.05	0.12	0.05	0.07	3.18	0.26	0.30	1.01	0.39	0.37	0.75	0.54	0.74	0.98	0.39	0.35	1.12	0.08
Xeno	Mean Cq	33.10	33.44	33.50	33.09	33.31	32.07	32.10	34.03	32.55	33.66	32.42	33.58	32.72	33.03	33.48	33.18	32.34	32.65
	SD of Cq	0.37	0.32	0.58	0.28	0.50	0.53	0.08	0.46	0.09	0.22	0.04	0.07	0.13	0.25	0.42	0.46	0.08	0.11

Three aliquots per mosquito pool homogenate were tested in triplicate qPCR reactions

The detection of *Ae. vigilax* mean Cq value and standard deviation (SD) are presented along with the log_10_ copy number per reaction

Inhibition was assessed by spiking each mosquito pool with Xeno inhibition control with mean Cq value and standard deviation presented

### Phylogenetics of Victorian *Ae. vigilax* specimens

**Fig. 4:**
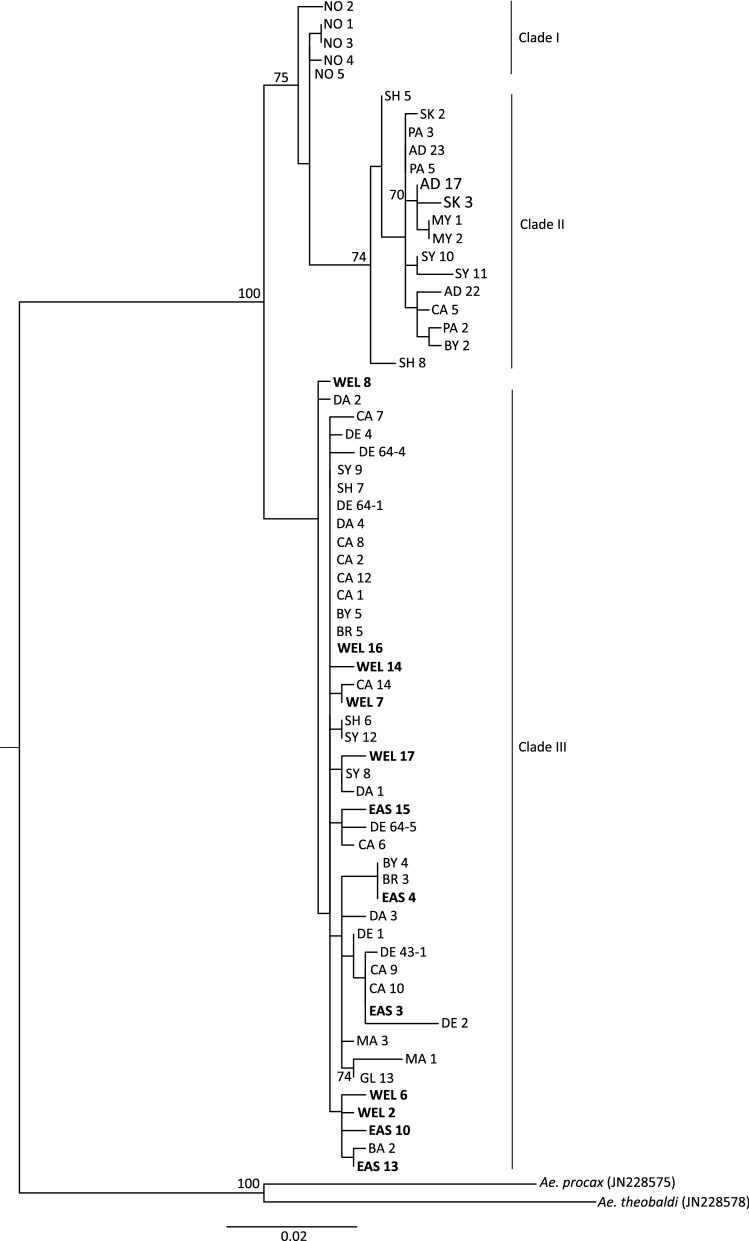
*Aedes vigilax* cytochrome c oxidase subunit 1, maximum-likelihood phylogenetic tree including sequences from the two capture locations within Victoria, denoted in boldface. All other sequences were obtained from Puslednik et al. [26]. Trap locations include Victoria, East Gippsland (EAS) and Wellington(WEL); South Australia, St Kilda (SK), Port Adelaide (PA), Adelaide (AD) and Mypolonga (MY); New South Wales, Byron Bay (BY), Port Stephens (PS), Sydney (SY), Shellharbour (SH) and Batemans Bay (BA); Queensland, Cairns (CA); Northern Territory, Darwin (DA); Western Australia, Derby (DE), Broome (BR), Goegrup Lake (GL) and Mandurah (MA); New Caledonia, Noumea (NO). Based on a 591-bp region of the COI gene. General time-reversible (GTR) substitution model was used with 1000 bootstrap replicates. Bootstrap proportions (BSP ≥ 70%) are indicated beside nodes. The number of nucleotide substitutions per site is represented by the scale bar. *Aedes procax* and *Ae. theobaldi* were used as an outgroup

**Fig. 5:**
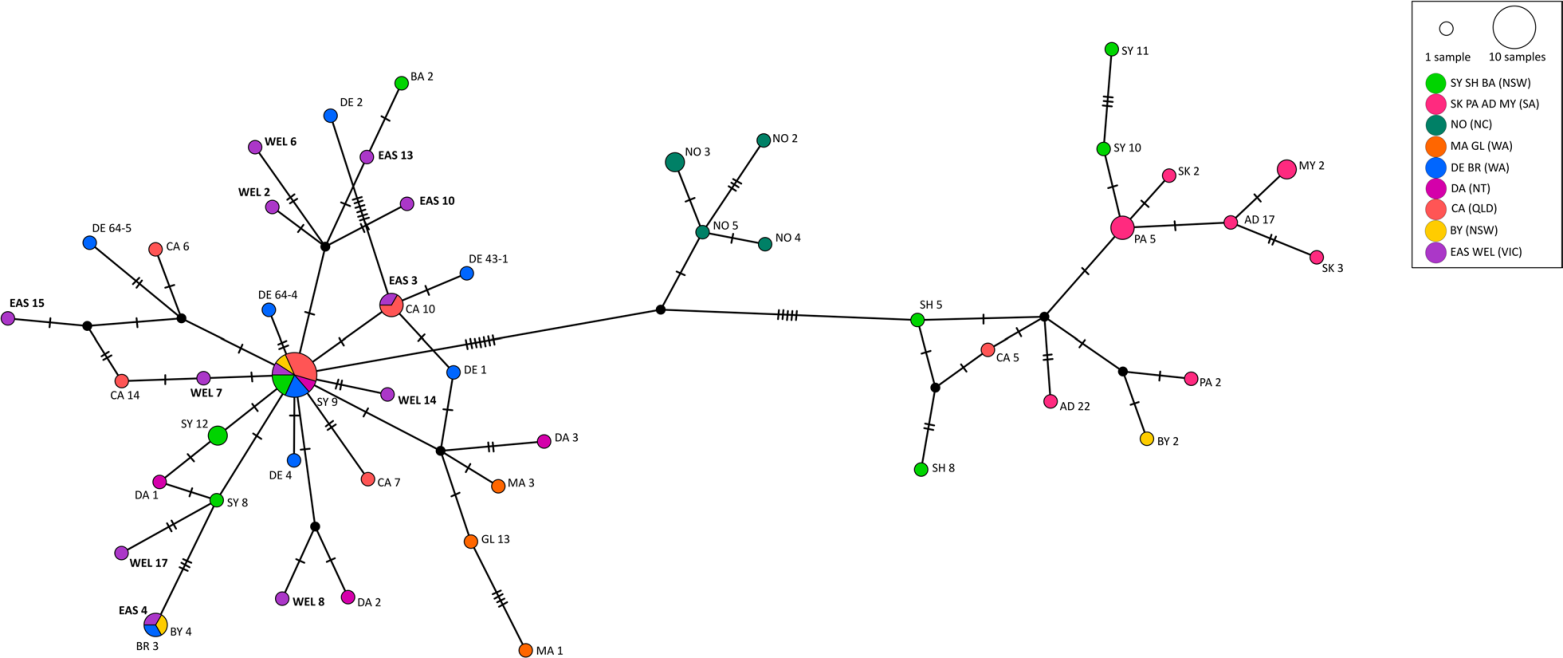
Cytochrome oxidase subunit I haplotype network of *Ae. vigilax* sampled across Australia and New Caledonia. Dashes along lines represent the number of mutations; color of each circle represents the geographical location the insect was collected from. Trap locations include Victoria, East Gippsland (EAS) and Wellington VIC (WEL); South Australia, St Kilda (SK), Port Adelaide (PA), Adelaide (AD) and Mypolonga (MY); New South Wales, Byron Bay (BY), Port Stephens (PS), Sydney (SY), Shellharbour (SH) and Batemans Bay (BA); Queensland, Cairns (CA); Northern Territory, Darwin (DA); Western Australia, Derby (DE), Broome (BR), Goegrup Lake (GL) and Mandurah (MA); New Caledonia, Noumea (NO)

*COI* sequences were successfully obtained for 12 *Ae. vigilax* from the Wellington (*n* = 7) and East Gippsland (*n* = 5) region in Victoria from 2018 to 2019. Phylogenetic analysis based on *COI* of *Ae. vigilax* from Australia and New Caledonia identified that all Victorian specimens cluster in clade III (Fig. 4). Intraspecies divergence based on *COI* among the three clades varied from 0.5 to 4.1% (average, 2.4%) (Table 2). Within the *Ae. vigilax* group collected in Victoria, the *COI* divergence ranged from 0.2 to 1.4% (average, 0.8%), with no distinct clustering of *Ae. vigilax* from Wellington or East Gippsland observed (Figs. 4 and 5). All 12 *Ae. vigilax* from Victoria were also found to have between 99 and 100% identity based on *COI* to *Ae. vigilax* that had been previously collected from Sydney (NSW), Shellharbour (NSW), Byron Bay (NSW), Cairns (QLD), Broome (WA), Darwin (WA) and Derby (WA) (Fig. 1). This high level of sequence similarity indicates potential gene flow between these regions.

**Table. 2:** Nucleotide sequence divergence among three *Ae. vigilax* clades, based on COI, alpha amylase and zinc finger gene

Gene		Clade I	Clade II	Clade III
COI	*Clade* I	0–0.68 (0.37)	1.02–2.37 (1.74)	1.18–2.71 (1.78)
	Clade II	1.02–2.37 (1.74)	0–1.18 (0.55)	0.51–4.06 (2.66)
	Clade III	1.18–2.71 (1.78)	0.51–4.06 (2.66)	0–2.71 (0.65)
AA	Clade I	0.6–1.33 (0.89)	0.36–2.17 (0.36)	0.48–2.9 (1.38)
	Clade II	0.36-2.17 (0.36)	0.24–2.54 (1.11)	0.12–3.86 (1.34)
	Clade III	0.48–2.9 (1.38)	0.12–3.86 (1.34)	0–3.14 (1.40)
ZF	Clade I	0–0.51 (0.28)	0.13–1.27 (0.69)	0.13–2.93 (0.83)
	Clade II	0.13–1.27 (0.69)	0.13–1.91 (0.79)	0-3.56 (0.88)
	Clade III	0.13–2.93 (0.83)	0–3.56 (0.88)	0–3.05 (0.88)

Across all sampling locations, both alpha amylase and the zinc finger (Additional file 4: Figure S1) region showed lower divergence on average compared to the *COI* region, with alpha amylase gene showing 0.1–3.9% (average, 1.3%) divergence (Table 2), and zinc finger 0–3.6% (average, 0.9%) (Table 2) between the clades. Upon comparison between the *Ae. vigilax* collected in Victoria, alpha amylase 0.4–1.3% (0.8%) and zinc finger 0–1.0% (average, 0.5%) showed a similar level of divergence to that of *COI* (Table 2). Phylogenetic analysis of alpha amylase and zinc finger failed to show any clear supported separation between the three clades (Additional file 4: Figure S1).

A haplotype network was used to further investigate the *COI* diversity, with three distinct groups observed. The haplotype network showed a star like pattern for clade III indicating population expansion. A high level of haplotype diversity was observed for *COI* [47] (Fig. 5, Table 3) as well as alpha amylase [63] and for the zinc finger [56] gene (Table 3). The highest level of nucleotide diversity was seen for the *COI* (Table 3) gene. Across all groups, the number of polymorphic sites was highest for clade III. For all clades, low nucleotide diversity but high haplotype diversity was observed, indicating only small differences occurring between the haplotypes. This is also highlighted in the *COI* haplotype networks with only a few nucleotide substitutions between haplotypes shown (Fig. 5). Neutrality test using Tajima’s D and Fu’s Fs were both negative and significant for *COI* clade III, with a similar result observed for the *Ae. vigilax* from Victoria, supporting the haplotype network and indicating past population expansion (Table 3).

**Table. 3:** Genetic diversity indices and neutrality tests for all *Ae. vigilax*, using COI, alpha amylase and the zinc finger sequence

	Sample size	Number of haplotypes	Haplotype diversity	No. polymorphic sites	Nucleotide diversity estimate		Tajima’s D	Fu’s Fs
					π	Θ		
COI								
All	66	47	0.969	66	0.0144	0.0245	− 1.400	− 2.928*
Clade I	5	4	0.9	5	0.0037	0.0040	− 0.560	− 0.578
Clade II	16	11	0.974	16	0.0064	0.0087	− 1.141	− 1.114
Clade III	45	20	0.903	35	0.0054	0.0147	− 2.278*	− 3.871*
Victorian	12	12	1	21	0.0069	0.0117	− 1.814*	− 2.337*
Alpha amylase								
All	66	64	0.9945	93	0.0126	0.0261	− 1.660	− 3.032*
Clade I	5	5	1	15	0.0089	0.0087	0.203	0.216
Clade II	16	16	1	43	0.0114	0.0167	− 1.344	− 1.675
Clade III	45	43	0.9989	76	0.0136	0.0261	− 1.395	− 2.668*
Victorian	12	11	0.9848	23	0.0080	0.0092	− 0.570	− 0.756
Zinc finger								
All	66	57	0.98868	72	0.0086	0.01978	− 1.922*	− 3.328*
Clade I	5	3	0.7	5	0.0028	0.00305	− 0.562	− 0.578
Clade II	16	16	1	28	0.00782	0.01112	− 1.223	− 1.359
Clade III	45	41	0.99394	56	0.00871	0.01688	− 1.709	− 2.560*
Victorian	12	10	0.95455	14	0.0052	0.00632	− 0.757	− 1.063

^*^P < 0.05

## Discussion

Recent years have seen an increase in the number of arboviral outbreaks occurring around the world as a result of the increased movement and establishment of significant vector species [50–52]. Complicating the detection of important vector species is that mosquito trapping can provide poor quality specimens and often many morphologically similar species that require highly specialized taxonomists to morphologically identify individual mosquitoes using a microscope. Molecular-based screening assays for the detection of mosquito species is an expanding area of research that has been successfully applied to detect a range of species [28, 31, 53]. Although *Ae. vigilax* occurs throughout most states of Australia [23] as well as in New Caledonia [23], Seychelles, Fiji [54], the Philippines, Indonesia, Malaysia, Taiwan and Timor-Leste [55], there is currently no specific molecular assay for the detection of this species. We have developed a specific and sensitive *Ae. vigilax* qPCR assay that can be used to screen whole mosquito traps and successfully detect a single *Ae. vigilax* among 1000 mosquitoes of other species. The specificity of the *Ae. vigilax* qPCR to detect all three clades of *Ae. vigilax* was assessed by in-silico analysis aligning multiple individuals from all three clades to the primer and probe sequences. A maximum of two mismatches across these three regions was seen for some individuals, but never more than one mismatch in the probe region indicating there would be successful binding, amplification and detection of all three clades. The *Ae. vigilax* assay was also assessed against 20 mosquito species covering five genera commonly collected in Victoria with no amplification detected, highlighting the specificity of the assay.

During mosquito season peak collections in excess of 20,000 mosquitoes can be collected in a single trap night in coastal areas, requiring subsampling of traps, increasing the likelihood that taxonomists may miss this important vector species [56]. The whole trap extraction methodology developed in this study is an accurate and efficient way to process mosquito traps of up to 1000 insects, which typically takes an experienced taxonomist up to 45 min to process. Previous studies have identified that whole trap extraction of arthropods can be negatively impacted by PCR inhibitors, affecting PCR amplification and detections [57, 58]. However, our results show that through the assessment of spiked exogenous internal positive controls, PCR inhibition can be negated for by proportionally increasing homogenization media, thereby ensuring detection sensitivity. The *Ae. vigilax* qPCR assay efficacy was tested by performing a six ten-fold serial dilution through a dynamic range of 2.08E^-1^ ng/µl to 2.08E^-6^ ng/µl (9.5 to 4.5 log_10_ copies/rxn) (Fig. 3) being tested. The assay was determined to have 94.9% efficiency. A seventh dilution was performed (3.5 log_10_ copies/rxn); however, not all replicates were detected, indicating the limit of detection for the assay.

Detection sensitivity of the assay was assessed by spiking pools of mosquitoes with a single *Ae. vigilax*. *Aedes vigilax* was successfully detected in all mosquito pool sizes of 200, 400, 600, 800 and 1000. There was no difference in the Cq vale for the detection of *Ae. vigilax* in pools sizes of 200, 400 and 800. However, there was a three Cq value increase for detections of *Ae. vigilax* in pools sizes of 600 and 1000. As majority traps collected during the state-wide surveillance program are below 1000 mosquitoes (Fig. 2), this highlights the suitability of this assay for routine screening of whole mosquito traps.

*Aedes vigilax* has been historically detected in Victoria on several occasions during the annual mosquito trapping program of the VADCP [11, 12]. However, in this study, we present the possible establishment of this mosquito species in two coastal regions of Victoria with this species being detected over 3 consecutive years. *Aedes vigilax* numbers over the 3 years typically peaked in mid-February to March (Fig. 2), with a similar trend observed in *Ae. vigilax* populations from South Australia [6] and New South Wales [59]. In general, the overall capture number of *Ae. vigilax* individuals was low compared to trapping in other states, which can capture ~ 20,000 individuals in a single trap night [56]. The lower number of *Ae. vigilax* collected in Victoria compared to the northern states may also be a result of factors such as tidal inundation events of breeding sites, rainfall [60] and temperature [61]. Additionally, many of the trapping sites are historical and were not moved in this study; hence, there may be more optimal trap locations closer to larval habitats. Additional methods could also be used to increase the chance of trapping *Ae. vigilax* in the area such as adding 1-octen-3-ol lures to the CO_2_-baited EVS traps, which has been shown to significantly increase collections of *Ae. vigilax* [63–64].

Phylogenetic analysis of *Ae. vigilax* identified that individuals collected from Victoria were positioned in clade III. *COI* sequences obtained from Victorian *Ae. vigilax* had up to 100% sequencing identity to those *Ae. vigilax* previously collected from a range of locations including Sydney (NSW), Shellharbour (NSW), Byron Bay (NSW), Cairns (QLD), Broome (WA), Darwin (WA) and Derby (WA) [23], indicating that one of these sites might have been the source of the original introduction to Victoria (Fig. 1). No phylogenetic separation was seen between *Ae. vigilax* collected from the two capture sites in Victoria, as has been observed for different locations across Australia [25]. This was not surprising because of the relative proximity between these two populations (closest trapping sites approximately 12 km apart). *Ae. vigilax* are capable of flying large distances, having been recorded to travel up to 9 km from their larval habitats [24], and previous reports of wind-borne dispersal of up to 50 km [55], highlight the potential of movement and mixing between these two Victorian populations. A high level of haplotype diversity was identified within the *COI* sequences of Victorian specimens with 12 haplotypes found (Table 3). The higher number of haplotypes and the shared sequence variation to the *Ae. vigilax* clade III have been seen in previous studies [23]. Neutrality test indicates that clade III has gone through a population expansion, which was also seen when investigating the Victorian *Ae. vigilax* individuals within this clade (Table 3). Puslednik et al. [26] identified that clade II and III subdivided many years ago and have developed separate lineages, followed by secondary contact and the current sympatric distribution of clade II and III [25]. This work is supported by a study performed by Shibani [25], which used microsatellite data to show there is no reproductive isolation between these *Ae. vigilax* clades [65].

*Aedes camptorhynchus* and *Ae. vigilax* are the primary vector species of RRV in coastal areas of Australia [10]. Currently, in Victoria *Ae. camptorhynchus* is the primary vector species of RRV along the coastline [14]. If *Ae. vigilax* successfully established in this area, it could result in changes to virus transmission dynamics. Previous studies have indicated that *Ae. vigilax* is a more competent vector of RRV compared to *Ae. camptorhynchus* [66]. *Aedes vigilax* has experimentally also been shown to be a more efficient vector compared with other mosquitoes in its ability to transmit BFV [67] and CHIKV [4]. If *Ae. vigilax* becomes more widespread and abundant across Victoria, this could increase the length of the RRV transmission season. Previous studies in South Australia have highlighted the occurrence of seasonal succession with *Ae. camptorhynchus* occurring from spring to early summer and *Ae. vigilax* occurring from mid-late summer and autumn [20]; with the establishment of *Ae. vigilax* in Victoria, a similar extended RRV transmission season could occur.

## Conclusions

Here we present the development of a specific and sensitive molecular assay for the detection of *Ae. vigilax* from whole mosquito traps. An optimized processing method was developed to screen up to 1000 mosquitoes without compromising detection sensitivity, allowing for rapid and specific detection of this species in surveillance samples. Further development and validation of this assay could allow the identification of the different life stages of *Ae. vigilax* and potentially enable detection of this mosquito species larvae in water samples, as has been outlined by previous studies [68]. *Aedes vigilax* collected in Victoria were seen to have a high sequence identity to those of clade III, with no separation seen between individuals from the two capture locations in Victoria, indicating mixing between these populations. Globally, recent years have seen the increased movement and establishment of mosquito species in new regions. At times these new establishments can result in increased disease burden in these areas [69]. This highlights the need to implement accurate and rapid testing techniques that can be used alongside traditional mosquito surveillance programs to detect the presences of significant mosquito vector species such as *Ae. vigilax*.

## Supplementary Information

**Additional file 1:Table S1.***Aedes vigilax* species information used in the multiple locus typing.

**Additional file 2:Table S2.***Aedes vigilax* NCBI reference numbers.

**Additional file 3:Table S3.***Aedes procax* and *Aedes theobaldi* NCBI reference numbers.

**Additional file 4:Figure S1.** Phylogenetic analysis of *Ae. vigilax* based on an 828-bp region of the alpha amalyse (**a**) and 786 bp region of the zinc finger (**b**) gene. Maximum-likelihood phylogenetic tree including sequences from the Wellington (WEL) and East Gippsland (EAS) capture locations within Victoria, denoted in boldface. All other sequences were obtained from Puslednik et al. [26]. General time-reversible (GTR) substitution model was used for both trees with 1000 bootstrap replicates. Bootstrap proportions (BSP ≥ 70%) are indicated beside nodes. The number of nucleotide substitutions per site is represented by the scale bar. *Aedes procax* and *Ae. theobald*i were used as an outgroup.

## Data Availability

All sequences generated in this study are publicly available from NCBI under accession numbers MW 351796-819 and MW542560-71.

## Abbreviations

Ae: *Aedes*
BFV: Barmah Forest virus
CHIKV: Chikungunya virus
CO_2_: Carbon dioxide
*COI*: Cytochrome c oxidase subunit 1
Cx: *Culex*
Cq: Cycle quantification
EVS: Encephalitis virus surveillance
GTR: general time-reversible
NSW: New South Wales
PCR: Polymerase chain reaction
QLD: Queensland
qPCR: Quantitative polymerase chain reaction
RFLP: Restriction fragment length polymorphism
RRV: Ross River virus
SD: Standard deviation
WA: Western Australia
