# Supplementary material for: Quantitative PCR assay for the detection of Aedes vigilax in mosquito trap collections containing large numbers of morphologically similar species and phylogenetic analysis of specimens collected in Victoria, Australia
Source: Parasit Vectors. 2021 Aug 28;14:434. doi: 10.1186/s13071-021-04923-y (PMC8401248; doi:10.1186/s13071-021-04923-y)
Supplement: Supplementary file 1 — Additional file 1:Table S1.Aedes vigilax species information used in the multiple locus typing. [file 13071_2021_4923_MOESM1_ESM.pdf]

Additional file 1. *Aedes vigilax* species information used in the multiple locus typing.

Ae. vigilax ID	Collection date	Collection location	GPS location		Sex
			Latitude	Longitude	
WEL 2	12/03/2019	Wellington	-38.05	147.58	F
EAS 3	15/04/2019	East Gippsland	-37.90	147.71	F
EAS 4	15/04/2019	East Gippsland	-37.90	147.71	F
WEL 6	22/04/2019	Wellington	-38.03	147.46	F
WEL 7	8/04/2019	Wellington	-38.06	147.24	F
WEL 8	22/04/2019	Wellington	-38.05	147.58	F
EAS 10	9/03/2018	East Gippsland	-37.89	147.68	F
EAS 13	21/03/2018	East Gippsland	-37.90	147.71	F
WEL 14	26/03/2018	Wellington	-38.33	147.24	F
EAS 15	29/03/2018	East Gippsland	-37.90	147.71	F
WEL 16	17/04/2018	Wellington	-38.05	147.56	F
WEL 17	23/04/2018	Wellington	-38.04	147.61	F
