# Supplementary material for: Quantitative PCR assay for the detection of Aedes vigilax in mosquito trap collections containing large numbers of morphologically similar species and phylogenetic analysis of specimens collected in Victoria, Australia
Source: Parasit Vectors. 2021 Aug 28;14:434. doi: 10.1186/s13071-021-04923-y (PMC8401248; doi:10.1186/s13071-021-04923-y)
Supplement: Supplementary file 2 — Additional file 2:Table S2.Aedes vigilax NCBI reference numbers. [file 13071_2021_4923_MOESM2_ESM.pdf]

**Additional file 2.** *Aedes vigilax* NCBI reference numbers.

| *Ae. vigilax* ID | Capture location | Australian state | Country | Accession number | | | Reference |
|---|---|---|---|---|---|---|---|
| | | | | COI | Alpha amalyse | Zinc finger | |
| EAS 3 | East Gippsland | Victoria | Australia | MW542561 | MW351797 | MW351809 | This study |
| EAS 4 | East Gippsland | Victoria | Australia | MW542562 | MW351798 | MW351810 | This study |
| EAS 10 | East Gippsland | Victoria | Australia | MW542566 | MW351802 | MW351814 | This study |
| EAS 13 | East Gippsland | Victoria | Australia | MW542567 | MW351803 | MW351815 | This study |
| EAS 15 | East Gippsland | Victoria | Australia | MW542569 | MW351805 | MW351817 | This study |
| WEL 2 | Wellington | Victoria | Australia | MW542560 | MW351796 | MW351808 | This study |
| WEL 6 | Wellington | Victoria | Australia | MW542563 | MW351799 | MW351811 | This study |
| WEL 7 | Wellington | Victoria | Australia | MW542564 | MW351800 | MW351812 | This study |
| WEL 8 | Wellington | Victoria | Australia | MW542565 | MW351801 | MW351813 | This study |
| WEL 14 | Wellington | Victoria | Australia | MW542568 | MW351804 | MW351816 | This study |
| WEL 16 | Wellington | Victoria | Australia | MW542570 | MW351806 | MW351818 | This study |
| WEL 17 | Wellington | Victoria | Australia | MW542571 | MW351807 | MW351819 | This study |
| CA 1 | Cairns | Queensland | Australia | JN228466 | JN228411 | JN228533 | Puslednik et al. 2012 |
| CA 10 | Cairns | Queensland | Australia | JN228467 | JN228418 | JN228540 | Puslednik et al. 2012 |
| CA 12 | Cairns | Queensland | Australia | JN228468 | JN228419 | JN228541 | Puslednik et al. 2012 |
| CA 14 | Cairns | Queensland | Australia | JN228469 | JN228420 | JN228542 | Puslednik et al. 2012 |
| CA 2 | Cairns | Queensland | Australia | JN228470 | JN228412 | JN228534 | Puslednik et al. 2012 |
| CA 5 | Cairns | Queensland | Australia | JN228471 | JN228413 | JN228535 | Puslednik et al. 2012 |
| CA 6 | Cairns | Queensland | Australia | JN228472 | JN228414 | JN228536 | Puslednik et al. 2012 |
| CA 7 | Cairns | Queensland | Australia | JN228473 | JN228415 | JN228537 | Puslednik et al. 2012 |
| CA 8 | Cairns | Queensland | Australia | JN228474 | JN228416 | JN228538 | Puslednik et al. 2012 |
| CA 9 | Cairns | Queensland | Australia | JN228475 | JN228417 | JN228539 | Puslednik et al. 2012 |
| DA 1 | Darwin | Western Australia | Australia | JN228476 | JN228421 | JN228543 | Puslednik et al. 2012 |
| DA 2 | Darwin | Western Australia | Australia | JN228477 | JN228422 | JN228544 | Puslednik et al. 2012 |
| DA 3 | Darwin | Western Australia | Australia | JN228478 | JN228423 | JN228545 | Puslednik et al. 2012 |
| DA 4 | Darwin | Western Australia | Australia | JN228479 | JN228424 | JN228546 | Puslednik et al. 2012 |
| DE 43-1 | Derby | Western Australia | Australia | JN228453 | JN228387 | JN228507 | Puslednik et al. 2012 |
| DE 64-1 | Derby | Western Australia | Australia | JN228454 | JN228388 | JN228508 | Puslednik et al. 2012 |
| DE 64-4 | Derby | Western Australia | Australia | JN228455 | JN228389 | JN228509 | Puslednik et al. 2012 |
| DE 64-5 | Derby | Western Australia | Australia | JN228456 | JN228390 | JN228510 | Puslednik et al. 2012 |
| DE 1 | Derby | Western Australia | Australia | JN228480 | JN228425 | JN228547 | Puslednik et al. 2012 |
| DE 2 | Derby | Western Australia | Australia | JN228481 | JN228426 | JN228548 | Puslednik et al. 2012 |
| DE 4 | Derby | Western Australia | Australia | JN228482 | JN228427 | JN228549 | Puslednik et al. 2012 |
| BR 3 | Broome | Western Australia | Australia | JN228461 | JN228528 | JN228406 | Puslednik et al. 2012 |
| BR 5 | Broome | Western Australia | Australia | JN228462 | JN228529 | JN228407 | Puslednik et al. 2012 |
| GL 13 | Goegrup Lake | Western Australia | Australia | JN228457 | JN228394 | JN228514 | Puslednik et al. 2012 |
| MA 1 | Mandura | Western Australia | Australia | JN228483 | JN228428 | JN228550 | Puslednik et al. 2012 |
| MA 3 | Mandura | Western Australia | Australia | JN228484 | JN228429 | JN228551 | Puslednik et al. 2012 |
| BY 2 | Byron Bay | New South Wales | Australia | JN228463 | JN228408 | JN228530 | Puslednik et al. 2012 |
| BY 4 | Byron Bay | New South Wales | Australia | JN228464 | JN228409 | JN228531 | Puslednik et al. 2012 |
| BY 5 | Byron Bay | New South Wales | Australia | JN228465 | JN228410 | JN228532 | Puslednik et al. 2012 |
| SY 10 | Sydney | New South Wales | Australia | JN228500 | JN228447 | JN228569 | Puslednik et al. 2012 |
| SY11 | Sydney | New South Wales | Australia | JN228501 | JN228448 | JN228570 | Puslednik et al. 2012 |
| SY12 | Sydney | New South Wales | Australia | JN228502 | JN228449 | JN228571 | Puslednik et al. 2012 |
| SY8 | Sydney | New South Wales | Australia | JN228503 | JN228445 | JN228567 | Puslednik et al. 2012 |
| SY 9 | Sydney | New South Wales | Australia | JN228504 | JN228446 | JN228568 | Puslednik et al. 2012 |
| SH 5 | Shellharbour | New South Wales | Australia | JN228496 | JN228441 | JN228563 | Puslednik et al. 2012 |
| SH 6 | Shellharbour | New South Wales | Australia | JN228497 | JN228442 | JN228564 | Puslednik et al. 2012 |
| SH 7 | Shellharbour | New South Wales | Australia | JN228498 | JN228443 | JN228565 | Puslednik et al. 2012 |
| SH 8 | Shellharbour | New South Wales | Australia | JN228499 | JN228444 | JN228566 | Puslednik et al. 2012 |
| BA 2 | Batemans Bay | New South Wales | Australia | JN228459 | JN228405 | JN228526 | Puslednik et al. 2012 |
| SK 2 | St Kilda | South Australia | Australia | JN228505 | JN228450 | JN228572 | Puslednik et al. 2012 |
| SK 3 | St Kilda | South Australia | Australia | JN228506 | JN228451 | JN228573 | Puslednik et al. 2012 |
| PA 2 | Port Adelaide | South Australia | Australia | JN228492 | JN228437 | JN228559 | Puslednik et al. 2012 |
| PA 3 | Port Adelaide | South Australia | Australia | JN228493 | JN228438 | JN228560 | Puslednik et al. 2012 |
| PA 5 | Port Adelaide | South Australia | Australia | JN 228494 | JN228439 | JN228561 | Puslednik et al. 2012 |
| AD 1 | Adelaide | South Australia | Australia | GQ143724 | JN228395 | JN228516 | Puslednik et al. 2012 |
| AD 3 | Adelaide | South Australia | Australia | GQ143725 | JN228396 | JN228517 | Puslednik et al. 2012 |
| AD 4 | Adelaide | South Australia | Australia | GQ143726 | JN228397 | JN228518 | Puslednik et al. 2012 |
| MY 1 | Mypolonga | South Australia | Australia | JN228485 | JN228430 | JN228552 | Puslednik et al. 2012 |
| MY 2 | Mypolonga | South Australia | Australia | JN228486 | JN228431 | JN228553 | Puslednik et al. 2012 |
| NO 1 | Noumea | | New Caledonia | JN228487 | JN228432 | JN228554 | Puslednik et al. 2012 |

| | | | | | | |
|---|---|---|---|---|---|---|
| NO 2 | Noumea | New Caledonia | JN228488 | JN228433 | JN228555 | Puslednik et al. 2012 |
| NO 3 | Noumea | New Caledonia | JN228489 | JN228434 | JN228556 | Puslednik et al. 2012 |
| NO 4 | Noumea | New Caledonia | JN228490 | JN228435 | JN228557 | Puslednik et al. 2012 |
| NO 5 | Noumea | New Caledonia | JN228491 | JN228436 | JN228558 | Puslednik et al. 2012 |
