# Supplementary material for: Quantitative PCR assay for the detection of Aedes vigilax in mosquito trap collections containing large numbers of morphologically similar species and phylogenetic analysis of specimens collected in Victoria, Australia
Source: Parasit Vectors. 2021 Aug 28;14:434. doi: 10.1186/s13071-021-04923-y (PMC8401248; doi:10.1186/s13071-021-04923-y)
Supplement: Supplementary file 3 — Additional file 3:Table S3.Aedes procax and Aedes theobaldi NCBI reference numbers. [file 13071_2021_4923_MOESM3_ESM.pdf]

Additional file 3. *Aedes procax* and *Aedes theobaldi* NCBI reference numbers.

Species	Accession number			Reference
	COI	Alpha amalyse	Zinc finger	
Aedes procax	JN228575	JN228574	JN228576	Puslednik et al. [26]
Aedes theobaldi	JN228578	JN228577	JN228579	Puslednik et al. [26]
