# Supplementary material for: Quantitative PCR assay for the detection of Aedes vigilax in mosquito trap collections containing large numbers of morphologically similar species and phylogenetic analysis of specimens collected in Victoria, Australia
Source: Parasit Vectors. 2021 Aug 28;14:434. doi: 10.1186/s13071-021-04923-y (PMC8401248; doi:10.1186/s13071-021-04923-y)
Supplement: Supplementary file 4 — Additional file 4:Figure S1. Phylogenetic analysis of Ae. vigilax based on an 828-bp region of the alpha amalyse (a) and 786 bp region of the zinc finger (b) gene. Maximum-likelihood phylogenetic tree including sequences from the Wellington (WEL) and East Gippsland (EAS) capture locations within Victoria, denoted in boldface. All other sequences were obtained from Puslednik et al. [26]. General time-reversible (GTR) substitution model was used for both trees with 1000 bootstrap replicates. Bootstrap proportions (BSP ≥ 70%) are indicated beside nodes. The number of nucleotide substitutions per site is represented by the scale bar. Aedes procax and Ae. theobaldi were used as an outgroup. [file 13071_2021_4923_MOESM4_ESM.pdf]

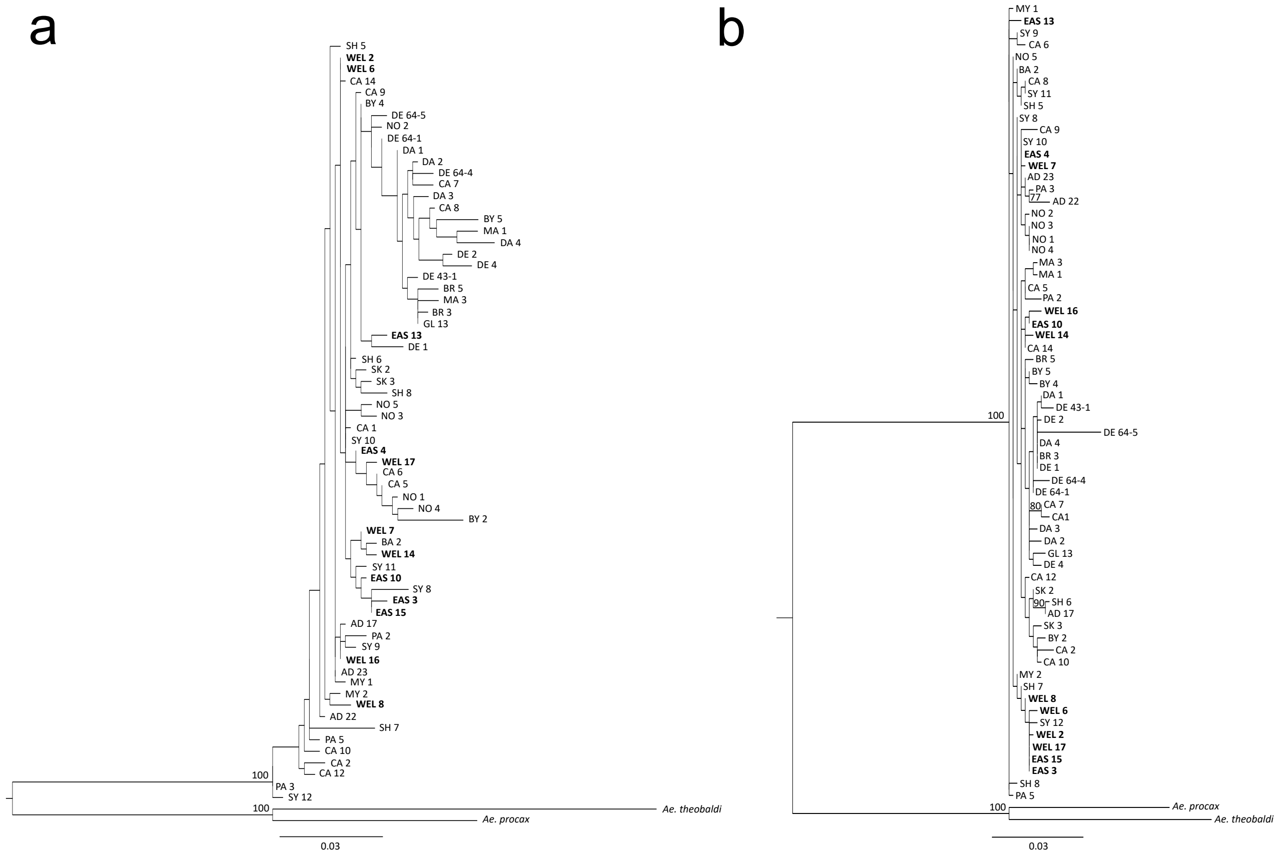

Additional file 4. Phylogenetic analysis of *Ae. vigilax* based on an 828 bp region of the alpha amalyse (a), and 786 bp region of the zinc finger (b) gene. Maximum -likelihood phylogenetic tree including sequences from the Wellington (WEL) and East Gippsland (EAS) capture locations within Victoria, denoted in boldface. All other sequences were obtained from Puslednik et al. [26]. General Time-Reversible (GTR) substitution model was used for both trees with 1,000 bootstrap replicates. Bootstrap proportions (BSP ≥ 70%) are indicated beside nodes. The number of nucleotide substitution per site is represented by the scale bar. *Aedes procax* and *Ae. theobaldi* were used as an out group.
